# HEALTH INSURANCE AND FAMILY CAREGIVERS: CAREGIVERS’ PERCEPTIONS OF COMMUNICATION BY SERVICE PROVIDERS

**DOI:** 10.1093/geroni/igae098.2413

**Published:** 2024-12-31

**Authors:** Alexa Balmuth, Adam Felts, Sophia Ashebir, Samantha Brady, Chaiwoo Lee, Lisa D’Ambrosio, Joseph Coughlin

**Affiliations:** Massachusetts Institute of Technology, Cambridge, Massachusetts, United States; Massachusetts Institute of Technology, Cambridge, Massachusetts, United States; Massachusetts Institute of Technology, Cambridge, Massachusetts, United States; MIT AgeLab, Cambridge, Massachusetts, United States; Massachusetts Institute of Technology, Cambridge, Massachusetts, United States; MIT AgeLab, Cambridge, Massachusetts, United States; MIT AgeLab, Cambridge, Massachusetts, United States

## Abstract

Health insurers not only play a key role in older adults’ access to medical care and services, but also interact with and, in some cases, provide benefits for caregivers of older family members. Additionally, family caregivers interact with their care recipient’s insurer to manage healthcare-related payments and when seeking information and support for their caregiving role. The relationship between health insurers and family caregivers can be just as important as their relationship with the plan member. Understanding how caregivers perceive and interact with their care recipient’s insurer is important to ensuring continuity of communication and care for older adults, as well as support for caregivers themselves. Paired with qualitative interviews, results from a survey of 244 current family caregivers in fall 2023, found that despite high levels of reported health insurance coverage among care recipients, many caregivers reported that their loved one’s health plans did not provide caregiving benefits, and many reported that they did not know if their care recipient’s health insurance offered any caregiving benefits. Results highlight caregivers’ perceptions and trust in health insurers – and how these vary across insurance types and providers. This session will provide implications for health service providers to improve communications with caregivers, including communications around their full range of offerings to caregivers and identifying caregivers’ preferred frequencies and modes of communication with insurers. Ultimately, this research aims to contribute to better health outcomes and quality of life for older adults and improved support for family caregivers.

